# Workforce preparation: the Biohealth computing model for Master and PhD students

**DOI:** 10.1186/1479-5876-12-S2-S11

**Published:** 2014-11-28

**Authors:** Marta Cascante, Pedro de Atauri, David Gomez-Cabrero, Peter Wagner, Josep Joan Centelles, Silvia Marin, Isaac Cano, Filip Velickovski, Igor Marin de Mas, Dieter Maier, Josep Roca, Philippe Sabatier

**Affiliations:** 1 Departament de Bioquimica i Biologia Molecular i IBUB, Facultat de Biologia, Universitat de Barcelona, 08028 Barcelona, Spain; 2 Hospital Clinic - Institut d'Investigacions Biomediques August Pi i Sunyer (IDIBAPS). Universitat de Barcelona, 08036 Barcelona, Spain; 3 BioHealth Computing Erasmus Mundus Master Program, University Joseph Fourier, Grenoble, France; 4 Unit of computational Medicine, Center for Molecular Medicine, Department of Medicine, Karolinska Institute and Karolinska University Hospital, SE-171 76 Stockholm, Sweden; 5 UCSD Division of Physiology, Pulmonary and Critical Care Medicine, University of California, San Diego, La Jolla, California, USA; 6 Department of eHealth, Barcelona Digital, Roc Boronat 117, 08017 Barcelona, Catalunya, Spain; 7 Biomax Informatics AG, Robert-Koch-Str. 2, Planegg, Germany; 8 Centro de Investigación en Red de Enfermedades Respiratorias (CibeRes), 07110 Palma de Mallorca, Spain; 9 Equipe environnement et prédiction de la santé des populations, Laboratoire TIMC (UMR 5525), CHU de Grenoble, Université Joseph Fourier, La Tronche, France

**Keywords:** Biomedical Research, Education, Integrated Health Care Systems, Master, PhD, Professionals, Systems Medicine, Training

## Abstract

The article addresses the strategic role of workforce preparation in the process of adoption of Systems Medicine as a driver of biomedical research in the new health paradigm. It reports on relevant initiatives, like CASyM, fostering Systems Medicine at EU level. The chapter focuses on the BioHealth Computing Program as a reference for multidisciplinary training of future systems-oriented researchers describing the productive interactions with the Synergy-COPD project.

## The new paradigm shift in healthcare

Discoveries in Health Sciences classically begin at "the bench", with basic research, and then progress to the clinical level, or patient's "bedside". Growing barriers and increasing complexities have made difficult to translate new knowledge to the bedside. But, scientists are increasingly aware that this "bench-to-bedside" approach shall really be a two-way system providing a fluent and efficient interplay between healthcare and basic biomedical research.

The purpose of the manuscript is to pull together the principal findings and recommendations of the various reports and publications concerning the paradigm shift in health taking into account an analysis of the changing nature of the interactions among health practice, research, and education. More specifically, we consider the implications for biomedical innovation from three perspectives: (i) Social context (Section: The social context); (ii) Scientific discipline (Section: Systems Medicine and workforce preparation); and (iii) Educational and training perspective (Section: Systems Medicine post-graduate education: the need for multidisciplinary collaboration). The latter addresses the question of what our global world should seek as objectives of biomedical education and innovation in the 21^st ^Century, recognizing that these must change significantly to address rapidly changing needs and priorities.

Achievement of major breakthroughs in biomedicine depends on rapid adaptation of training schemes and on long-term investment in cutting-edge research. To this end, we analyze the Biohealth Computing program for Master and PhD students, as an example of educational and training program aiming to produce translational researchers aligned with the new health paradigm. Finally we suggest a roadmap to the future: a series of recommendations and actions aimed at transforming practice, research, and education, with the fundamental objective of sustaining and enhancing our capacity for biomedical innovation which constitutes a key element for economic prosperity and social well-being.

Systems thinking approach is not new to biomedicine, having been historically applied in the physiological sciences. However, it had to be abandoned because of the lack of necessary data and tools. Today, the revolutions in molecular biology and in Information and Communication Technologies (ICT) constitute key enablers to re-assess old problems under the new health paradigm.

## The social context

During few exhilarating decades, in the middle of the twentieth century, it seemed the world might have a reprieve from some major diseases. But, during last two decades of the last century, the concern about the epidemics of Non-Communicable Diseases (NCDs) increasingly grew [1-4]. The need for novel healthcare approaches for chronic patients triggered the Chronic Care model first formulated in early 2000 [5]. It was soon adopted by the World Health Organization (WHO) through the Innovative Care for Chronic Conditions (ICCC) initiative [5-9]. In September 2011, the United Nations (UN) General Assembly formally acknowledged the impact of the NCDs epidemics and the need for reshaping health systems worldwide [1,4] toward adoption of the Chronic Care model. Further studies on health trends [10] confirm NCDs as: (1) leading cause of healthcare burden and mortality in the world; (2) increasing in prevalence; (3) extremely expensive with conventional healthcare approaches; and, (4) an under-appreciated cause of poverty and hindered economic development [1]. NCDs are multi-factorial in nature and involve complex gene-environment interactions [4]. Environmental factors, unhealthy life-styles and increased life expectancy (ageing), intrinsic host responses, such as local and systemic inflammation [11], epigenetic changes [12] and decoupling of basic regulatory mechanisms with impact on bioenergetics, immune responses and remodeling [12] may play significant roles in the initiation and persistence of NCDs and associated co-morbidities. Accordingly, the management of NCDs should move towards holistic approaches aiming at tackling all components of their complexities through deployment of integrated care services, as proposed by the Chronic Care model [5-9].

The on-going transition toward an integrated care approach in several EU regions *(European Innovation Partnership for Active and Healthy Ageing, EI-AHA) *[13] is fostered by three main driving forces: (i) the burden imposed by the epidemics of NCDs [10]; (ii) the need for generating efficiencies allowing further investments on innovation without increasing overall health costs; and, last but not least important, (iii) the paradigm change in the understanding of underlying mechanisms of NCDs [13-16]. A commonality of these three driving forces is the need for efficient and intensive use of Information and Communication Technologies (ICT) to support novel Integrated Care Services (ICS) for chronic patients [17]. The convergence between ICS and novel systems-oriented biomedical research is a pivotal unmet need for an effective deployment of 4P *(Predictive, Preventive, Personalized and Participatory) *medicine, as conceived in the Synergy-COPD project.

## Systems medicine and workforce preparation

### The concept of Systems Medicine

As defined by Federow and Gostin [18], stemming from Systems Biology, Systems Medicine focuses on biomedical research incorporating interactions between all components of health and disease. Systems Medicine is an approach that aims at understanding the multitude of non-linear dynamic interactions among components of a living system and their interplay with the environment. In this scenario, biomedical questions are addressed through integrating experiments in iterative cycles using computational modeling [13-16,19,20] to capture emergent properties that arise from dynamic interactions at systems level and cannot be inferred with a reductionist approach. Systems Medicine is proposing an innovative approach to our understanding of disease mechanisms that is contributing to build-up patient-based predictive medicine with important implications on future strategies for patient management.

### Holistic focus and use of models for system description and prediction of the system response to interventions

Under the new paradigm of Systems Medicine, a holistic approach requires the challenge of data integration (-omics, physiologic, clinical and environmental) as well as use of computer modeling for knowledge generation. This challenge can only be successfully achieved with the incorporation of tools traditionally restricted to physics, mathematics and bioinformatics to the classical arsenal of molecular biology and physiology with which biomedicine researchers and physicians have been mainly trained during the last decades [21-24].

### Systems Medicine of chronic disorders

It is well accepted that NCDs are caused by complex gene-environment interactions and they show clear socio-economic determinants. Moreover, it is well known that several chronic diseases are often present in a given patient as co-morbid disorders. Both analysis and management of NCDs with a reductionist approach does not appear to be efficient.

Recent advances in systems biology including network analysis are opening new avenues suggesting that commonly clustered co-morbid conditions may share abnormal regulation of pivotal metabolic pathways. The promising scenario generated by Systems Medicine may contribute to markedly enhance chronic patient's management based on better knowledge of bio-pathological mechanisms explaining disease occurrence and progress, as well as underlying factors determining clustering of co-morbid disorders.

The enhanced knowledge should facilitate future early prevention strategies with a personalized approach that may likely modulate chronic disease progress improving patient's prognosis. We understand that this constellation of forces should prompt a future convergence between deployment of integrated care and predictive medicine of NCDs wherein novel ICT applications can play an enabling role.

### A novel approach to disease taxonomy: from symptoms and individual biomarkers to dysfunctional biological networks

Disease diagnosis is traditionally based on assessment of characteristic symptoms/signs and individual biomarkers that lead to disease diagnosis. It is of note that major disease taxonomies, as well as therapies, are often organ-based [18]. Instead, Systems Medicine shows high potential to build-up novel and more functional disease taxonomies based on the achievements of network analysis producing knowledge on underlying mechanisms of diseases. The novel approach routed on understanding dysfunctional biological networks as disease mechanisms may open the way to innovative preventive and therapeutic strategies [15,16,18,20]. In this context, the goal of 4P medicine is to identify biological/environmental markers in order to enrol individuals at high risk for developing a disease in early detection and prevention tracks.

## Systems medicine post-graduate education: the need for multidisciplinary collaboration

A major bottleneck for the development of Systems Medicine (and 4P Medicine) is lack of availability of experienced biomedical researchers with strong background in bioinformatics/mathematics that are able to cope with the current needs for both knowledge and data integration. Before the existence of multi-disciplinary educational programs there was not a consensus if it was "*better to start with people from a domain of science and try to give them data management skills, or start with people from a computer science background and try to give them domain skills*" [25]. However, in the last decade new multi-disciplinary educational programs are being developed opening the avenue for new Systems Medicine-driven educational and training profiles. It is our understanding that biomedical research can greatly benefit from the rapid emergence of the inter-disciplinary training programs bridging biological sciences, applied mathematics and medicine.

By training we consider both the *training as the development of skills *(e.g. learning as the use of tools and databases or a experimental technique) and the *training as the learning of knowledge and understanding of fundamental concepts *(e.g. through theoretical courses) [26]. The end-users of the training are future researchers aiming to work on the Systems Medicine paradigm. Among them, we may differentiate two profiles: those with major interest in the clinical/biological field and those more focused on the method-development. In both cases, training with a Systems Medicine approach, applies. Moreover, two important aspects must be considered in the training programs in Systems Medicine:

• Training across academic disciplines: Medicine, Physiology, Biotechnology, Mathematics, Physics, Bioinformatics, etc...

• Training new generations to be able to identify those areas where a systems approach will be most suitable to better address clinical questions, solve clinical problems or contribute to make health sector economically sustainable. For example, the Summer School's experience within the Biohealth Computing program has shown that Chronic Obstructive Pulmonary Disease (COPD) as a case study is one of these areas wherein a systems approach can be highly productive.

### The CASyM initiative

Recently, laudable efforts are being made worldwide to implement new multidisciplinary initiatives with a systems medicine approach. To mention just two examples: The Coordinated Action for Systems Medicine (CASyM) at EU level [27] and the MD/MS program in Systems Medicine recently launched by Georgetown University in USA [28]. CASyM was initiated in 2012, engaging 22 partners from 11 countries, with the mission of developing European wide implementation strategy for Systems Medicine that should integrate multiple elements: from clinical trials, drug development, public health and to medical economics initiatives.

The CASyM roadmap aims to identify clinical questions/problems that can be better addressed/solved using a systems approach. CASyM aims to engage relevant stakeholders in the implementation of research programs. It is also within CASyM's goals to contribute to the development of multidisciplinary training concepts toward a sustainable Systems Medicine implementation strategy for Europe.

The success of Systems Medicine will depend strongly on the extent to which the leaders accomplish the creation of the environment that researchers need to develop an understanding among different working cultures, and manage also to implement strategies that integrate these cultures into shared working practices. Senior academics can have the initial vision for a project in Systems Medicine, but it will inevitably be the junior researchers, and their networking with peers, who will develop the collaborative relations that eventually will deliver results. The infrastructure and working environment for the Systems Medicine must therefore be one that generates and fuels interaction at all levels. Junior researchers need to be encouraged and supported to commit time to such relations, and the familiarity and knowledge that they gain in the other discipline needs to be recognized.

### Origin and mission of the BioHealth Computing (BioHC) Post-Graduate School

The BHC Post-Graduate School (i.e. Masters and PhD programs) was conceived in 2010 to foster multidisciplinary training of researchers with a Systems Medicine approach aiming at fostering European competitiveness in biomedical sciences as well, as clinical research, and public health services in the 21^st ^century. The program was designed to be outward looking, opened to world-class research, ranging from basic science to clinical practice and industrial products. Considering that a major educational initiative in Systems Medicine will require a broad base of disciplines associating Medical and Life sciences but also Chemistry, Physics and Computational mathematics. Five European universities (Grenoble, Barcelona, Torino, Maastricht and Cluj-Napoca) decided to join efforts in the BioHealth Computing consortium (BioHC). The BioHC consortium associates a unique network of public-private organizations, including university hospital, bio-parks, industries and non-European academia from Asia and Latin-America, to create opportunities and to catalyze the development of Systems Medicine in Higher Education and Research. The BioHC consortium draws on the large expertise and technological resources available at the partner institutions to provide Master and PhD students with the tools necessary to address real world problems in a creative, interdisciplinary team setting.

The aim of the BioHC consortium is to bridge the double gap between bedside, bench and engineering researches, and between academic lab, industrial R&D and healthcare services, by implementing a multidisciplinary and inter-institutional Post-Graduate program (Master and PhD). The BioHC School has been implemented in 2011 at Master level and in 2014 at Doctoral level. The BioHC School trains students to extend medical technologies and advance pharmaceutical R&D and applications by leveraging advanced mathematical and computational methods in medical research and practice. The BioHC students gain a deeper understanding of the dynamics of pathophysiological mechanisms and facilitate their early diagnosis. The BioHC program implements interdisciplinary approaches in medical research and clinical practice. Systemic modeling and use of simulation tools and IT are a must to overcome technological obstacles described above and to develop a Product Lifecycle Management (PLM) approach in Medtech and Pharmatech which should have important consequences for the structure and functioning of the biopharmaceutical innovation system with contributions of BioHC in the increase of industrial competitiveness and reduction of overall healthcare costs.

### BioHC Post-Graduate program

Currently, the education and training of Systems Medicine researchers begins at postgraduate level. Since Systems Medicine is a new area of research, it is difficult to single out any one model of education and training as superior at this early stage. The tendency reflects the importance of mastering a parent discipline before moving on to a more wide ranging training. Nonetheless, rooting in a parent discipline allows the acquisition of a thorough understanding of the scientific method as well as providing a safe return path. The importance of this should not be underestimated, especially in light of the currently uncertainty of career opportunities in cross-discipline research. Systems researchers should be given opportunities to maintain their expertise in their parent discipline, for example by provision of protected time to pursue mono-disciplinary research. The BioHC Partner universities have introduced a 2^nd ^year Master and 3-year PhD program in Systems medicine. In countries like Spain wherein most undergraduate studies have a 4-year length, the Master degree has only 1-year duration instead of 2-years. In this case, the graduate students can enter directly into the BioHC Program.

#### Required profiles and parent disciplines

As mentioned, the mastering a parent discipline before enrolling the BioHC Master Course program (2^nd ^year) is required. Parent disciplines learned by the students (during the 1^st ^year of the Ms) have to fit with one of the four dominant academic tracks described below. A mobility scheme provides for at least two locations in the same track. The four tracks are:

**Clinical research**. The objectives of this track are: (a) Enhance research skills for residents from the EU and other areas (PhD); (b) Prepare Allied Health Care Professionals for research activities; (c) Professional development for physicians and Allied Health Care Professionals.

**Molecular biotechnology**. The applicants have to master the techniques for the development of biotechnological applications. The Biotechnology is the use of microorganisms, eukaryotic cells or biological substances, such as enzymes, to perform specific industrial or manufacturing processes.

**Environmental health**. The applicants have to know the principles of exposure assessment, and its role in environmental hygiene, epidemiology, toxicology and risk assessment. After completing this course the student has gained understanding of the importance and complexity of exposure assessment..

**Computational mathematics**. The students must know the mathematical methods and computational tools available for the dynamic modeling, analysis and validation, of biological regulatory networks. Lectures focus on qualitative and analytical aspects of mathematical modeling, as well as on real applications.

The BioHC Erasmus Mundus Master Program (60 ECTS) encompasses four well defined periods: (i) Integrative period of Master students at the Summer School (6 ECTS), (ii) First semester in one of the partner universities (24 ECTS); (iii) Second semester in a different partner university (24 ECTS); and, (iv) Consolidation period at the Summer School (6 ECTS). The BioHC program anticipates that upon receiving their Master's degree, or after a PhD program, graduates will work R&D department in industries or academia for developing innovative diagnostic, therapeutic or healthcare products.

#### Main principles of the BioHC program

Four major principles are guiding the BioHC program:

***Problem-based teaching ***is an approach in which small groups of students deal with previously constructed problems. Under supervision of BioHC Staff members and Invited Professors, the students build-up learning pathways to the point of producing syntheses and new knowledge. The problems are descriptions of a phenomenon or event to be analyzed by the group using prior knowledge. From this, the students seek to understand the underlying processes, and questions arise. This process consolidates the learning objectives and serve as the individual and group study content. Following this, the students check whether new information leads to an understanding of the problem, and discuss it again. The tutor takes on the role of facilitator, thereby stimulating the process and reflection on it.

***Student-centered learning***. It is well established that the teacher should provide guidance, tools and tasks, but the student needs to find for him/her self the solutions. This process generates self-confidence. The students show similar learning pattern despite the speed and outcomes may be different among them, depending upon skills. The research training is spread over two semesters, and the curriculum, also distributed over two semesters, is defined from the research topic assigned to the students. Research topics assigned to students addressed complex problems involving an interdisciplinary approach. The student's projects are led by two supervisors from two different scientific areas. The aim of this Joint Research Project is to give to BioHC student opportunity to develop a translational and multidisciplicary approach.

For the assignment of one subject to one student, it is anticipated that the topics will be relevant to the parent discipline of the student, and will address a question of importance for the student. Rather than dissecting complex disease processes and studying the individual parts of living systems, the BioHC program focuses on understanding the complete system and the underlying interactions of all the forces that make up that system. The curriculum of the students includes core courses related to the parent discipline of the student, and elective courses related to complementary disciplines. One student enrolled primarily in Clinical research track can follow secondarily courses given in the Computational mathematics track, to acquire skills needed for modeling biological interactions embedded in a pathological process. The main learning output of this student-centered learning is the students' ability to work on a joint research program. There was increased motivation, leadership development and team working from the students. This was translated through their written work, seminars and portfolio preparation. The evaluation process for these experiences presupposes well-founded practices that express the views of the subjects involved: self-assessment and observer assessment.

***Mobility path and double degrees*. **BioHC Partner universities have decided, in 2011, to seek to provide a solid grounding in Systems Medicine, for their students not only in the form of local education but also by actively encouraging them to broaden their horizons by mobility path. Based on their research topics, BioHC students follow a mobility scheme where they attend two labs of the participating universities. Each student's project is co-supervised by two PI from two Partner universities, and the students must spend at least 1 semester in each university. The projects can associate also an industry or a partner hospital and one university. The supervisors, from primary and secondary hosting Institutions, constitute the candidate's Supervisory Committee. The two supervisors have equal responsibilities and cooperate with student in the choice of training activities, including the definition of mobility choices, and supervise their research. Where possible, evaluation is held on the occasion of a conference or similar event organised by any of the BioHC Partners.

The BioHC Program meets the specifications of the Erasmus Mundus from the European Commission (EACEA): (i) mobility path between at least the two Partner Institutions; (ii) and giving rise to double degree after only one defence at one of the two universities of attachment, according to the national regulations for Academic Degrees (Master and PhD) and University's evaluations. Erasmus Mundus is open to both European and non-European students, and there are generous scholarships and funding opportunities available.

### BioHC Thematic Schools

The BioHC program has successfully developed the Summer School since 2012 and proposes additional Thematic Schools (TS) on specific ICT domains that may be a useful bridge between applied mathematicians and bio-scientists.

#### Interactive Training Initiative (Synergy-BioHC Summer School)

The Interactive Training Initiative (ITI) is proposed to the Ms students during the BioHC Summer School, at the opening of the BioHC Master Courses. This program brings together students, scientists and health professionals, in the International Campus of the Archamps Technopole (Geneva Lake area), to work on clinical findings in all phases of the "bench to bedside" process. The ITI provides students with concepts, methodology and tools to respond to challenging situation in Clinical and Life Sciences. Content covers the fundamentals of physiological and clinical processes, along with core medical principles, clinical research methods, and clinical trials design, as well as basics of applied mathematics and computing. The program culminates in a capstone design-project in which students work in interdisciplinary teams co-advised by faculty members and investigators from industries and hospitals. Initial focus areas include healthcare, therapeutic design and delivery, construction of medical devices, tissue engineering and regenerative medicine. A title of example, in Chronic Obstructive Pulmonary Disease (COPD), BioHC Master projects are focussed on studying network perturbations and key players that result in fatal Lung Cancer and COPD progression. Coursework material includes translational aspects of therapeutic science and principles of engineering design.

Introductory conferences on Systems medicine and P4 Medicine are given during each morning, by internationally recognized scientists, and students are invited, during each afternoon and evening, to work together on one R&D project, by group of 5-6 persons coming from mixed educations and skills. This R&D project is relevant to modern-day translational research in the field of Public Health. The focus of the ITI is on problem-based approach through team working: (i) realizing a goal; (ii) on utilizing the available expertise of the team and of the professional and teaching staff. Through coursework and collaborative research, the ITI program aims to enable candidates to combine experimental and theoretical approaches to develop physical and quantitative models of biological processes.

#### School initiatives currently in the design phase

The high volumes of data, the variety of data sources and the time-criticality of the analyses of biomedical and clinical problems are opening an opportunity window for big-data analytics. The BioHC partner universities are developing decision-support technological tools for analysis of huge amounts of data to help professionals, healthcare providers, payers and biomedical industry. To this end, big data analytics and computational modeling have been identified as relevant topics for thematic open school activities (Spring or Winter programs) following the principles of the BioHC program that should be addressed to both Master and PhD students.

## Conclusions

Convergence between Systems Medicine research and adoption of Integrated Care Services constitutes a fundamental strategic move toward consolidation of the new health paradigm that will make 4P medicine a reality. However, a key pillar for the transition phase is the preparation of the workforce with a multidisciplinary orientation. The BioHC program described in the current chapter has evolved through a successful learning period constituting a practical example of a systems-oriented training initiative that has consolidated productive interactions during the life span of the Synergy-COPD project.

## Competing interests

DM is employed at Biomax Informatics AG. The other authors declare that they have no competing interests.

## Authors' contributions

MC, PdA, PW, DM, DG-C, JR and PS conceived and designed the manuscript. MC, JR and PS write the first draft of the manuscript. MC, PdA, DG-C, JJC, SM, JR and PS contributed to the writing of the manuscript. PW, IC, FV, IMdM and DM revised the article critically for important intellectual content and gave final approval of the version to be submitted and any revised version.

## Declaration

Publication of this article has been funded by the Synergy-COPD European project (FP7-ICT-270086). The opinions expressed in this paper are those of the authors and are not necessarily those of Synergy-COPD project's partners or the European Commission.

This article has been published as part of *Journal of Translational Medicine *Volume 12 Supplement 2, 2014: Systems medicine in chronic diseases: COPD as a use case. The full contents of the supplement are available online at http://www.translational-medicine.com/supplements/12/S2.
